# Influence of Choice of Null Network on Small-World Parameters of Structural Correlation Networks

**DOI:** 10.1371/journal.pone.0067354

**Published:** 2013-06-28

**Authors:** S. M. Hadi Hosseini, Shelli R. Kesler

**Affiliations:** 1 Department of Psychiatry and Behavioral Sciences, Stanford University School of Medicine, Stanford, California, United States of America; 2 Stanford Cancer Institute, Palo Alto, California, United States of America; University of Namur, Belgium

## Abstract

In recent years, coordinated variations in brain morphology (e.g., volume, thickness) have been employed as a measure of structural association between brain regions to infer large-scale structural correlation networks. Recent evidence suggests that brain networks constructed in this manner are inherently more clustered than random networks of the same size and degree. Thus, null networks constructed by randomizing topology are not a good choice for benchmarking small-world parameters of these networks. In the present report, we investigated the influence of choice of null networks on small-world parameters of gray matter correlation networks in healthy individuals and survivors of acute lymphoblastic leukemia. Three types of null networks were studied: 1) networks constructed by topology randomization (TOP), 2) networks matched to the distributional properties of the observed covariance matrix (HQS), and 3) networks generated from correlation of randomized input data (COR). The results revealed that the choice of null network not only influences the estimated small-world parameters, it also influences the results of between-group differences in small-world parameters. In addition, at higher network densities, the choice of null network influences the direction of group differences in network measures. Our data suggest that the choice of null network is quite crucial for interpretation of group differences in small-world parameters of structural correlation networks. We argue that none of the available null models is perfect for estimation of small-world parameters for correlation networks and the relative strengths and weaknesses of the selected model should be carefully considered with respect to obtained network measures.

## Introduction

In recent years, coordinated variations in brain morphology (e.g. volume, thickness, surface area) have been employed as a measure of structural association between brain regions to infer large-scale structural correlation networks [1]–[20]. Alterations in the arrangements of these large-scale structural correlation networks have been associated with normal aging [3], [9], [10], multiple sclerosis [6], Alzheimer’s disease [4], [12], schizophrenia [8] and epilepsy [2], [15].

Structural correlation networks constructed in this manner are usually represented by a set of nodes that correspond to brain regions and a set of edges (connections) that correspond to statistical correlations in morphometric values between regions, across individuals [5], [11]. These networks have been shown to follow small-world architecture in healthy individuals [2]–[4], [6], [8], [17]; an architecture that provides optimal balance between local and global information processing in the network [21]–[24] and has been observed in biological and social networks [25], [26].

The small-worldness of a network is often characterized by two key metrics: the clustering coefficient *C* and the characteristic path length *L* of the network. The clustering coefficient of a node is a measure of the number of edges that exist between its nearest neighbors (nodes that are directly connected) [21], [27]. The clustering coefficient of a network is thus the average of clustering coefficients across nodes and is a measure of network segregation [28]. The characteristic path length of a network is the average shortest path length between all pairs of nodes in the network and is the most commonly used measure of network integration [28], [29]. To evaluate the small-world topology of the brain networks, these topological parameters must be benchmarked against corresponding mean values of a null random graph [30]–[33]. Thus, the small-worldness index of a network is obtained as SW = [C/C_null_]/[L/L_null_] where C_null_ and L_null_ are the mean clustering coefficient and the characteristic path length of the *m* null random networks, respectively [22]. In a small-world network, the clustering coefficient is significantly higher than that of random networks (*C/C*_null_ ratio greater than 1) while the characteristic path length is comparable to random networks (*L/L*_null_ ratio close to 1) resulting in a small world index of SW >1. Obviously, the small-world index of a network is largely affected by the choice of null network [32], [33].

In the present study, we investigated the effects of choice of null networks on small-world properties of structural correlation networks. The null networks are usually constructed using rewiring algorithms that preserve the topology of the graphs; i.e. random graphs with the same number of nodes, total edges and degree distribution as the network of interest [30], [31]. However, recent evidence suggests that networks constructed from correlations are inherently more clustered than random networks of the same size and degree and correlation transitivity induces an additive small-world organization to the network [33]. The correlation transitivity effect suggests that the existence of a strong positive correlation between regions A and B as well as B and C would result in a strong positive correlation between regions A and C. This effect induces an inflated clustering in correlation networks. Thus, constructing networks from correlation of a set of random vectors would also lead to a network with small-world characteristic rather than a random network. Therefore, topology randomization overestimates the small-worldness of correlation networks by annihilating the transitive structure induced by correlation transitivity. To overcome this limitation, Zalesky and colleagues [33] proposed generating null covariance matrices that are matched to the distributional properties of the observed covariance matrix using the Hirschberger-Qi-Steuer (HQS) algorithm [34]. The suggested null network is believed to solely annihilate intrinsic structure in the empirical network and does not affect the transitive structure (i.e. structure induced by correlation transitivity). Thus, it gives a more conservative estimate of normalized clustering coefficient of correlation networks relative to random graphs. However, compared to topology-preserving methods, the HQS method underestimates the relative characteristic path length of the network [33]. There is still no evidence on how the choice of null networks affects the small-world parameters of empirical structural correlation networks.

In this report, we investigated the influence of choice of null network on small-world index of gray matter correlation networks in healthy individuals and survivors of acute lymphoblastic leukemia (ALL), a population that we previously demonstrated to have altered large-scale brain networks [19]. We studied three types of null networks: 1) networks constructed by topology randomization (TOP) [30], [31], 2) networks matched to the distributional properties of the observed covariance matrix using Hirschberger-Qi-Steuer algorithm (HQS) [33], [34], and 3) networks generated from correlation of randomized input data (COR). The latter is an intuitive way of generating null networks for benchmarking correlation networks by applying the same network construction procedure on the randomized input data. We studied the influence of choice of null networks on the small-world parameters of the networks at group-level as well as on the significance of between-group differences in small-world parameters. In addition, we investigated whether differences between null networks are affected by the regime of binarization threshold. We discussed the pros and cons of different null networks and qualitatively discuss potential solutions that need to be formulated and validated in future studies.

## Materials and Methods

### Participants

The detailed procedures of participants, data acquisition and preprocessing are published elsewhere [35]. In summary, 28 children and adolescents with a history of ALL (age 5.0–19.8 years old) who had completed all anti-cancer treatments for at least 6 months as well as 31 healthy controls (HC) (age 4.1–18.4 years old), matched for age, gender, maternal education level and minority status, were recruited.

### Ethics Statement

The study was approved by the Stanford University Institutional Review Board and the Stanford Cancer Institute’s Scientific Review Board and written informed consent was obtained from adult participants or from the parent/legal guardian of minor participants and assent was obtained from participants age 8 years and older per Stanford University’s regulations. We could not make this data available to public because of privacy issues (i.e. participants were not consented for inclusion in a public database).

### MRI Data Acquisition and Preprocessing

High resolution, 3D spoiled gradient recall MR images were obtained using a 3 Tesla GE Signa whole body scanner (GE Medical Systems, Milwaukee, WI) with the following parameters: repetition time = 6.436 ms, echo time = 2.064 ms, flip angle = 15°, number of excitation = 3, matrix size = 256×256 voxels, field of view = 220, slice thickness = 1.5 mm, 124 contiguous slices. To extract individual gray matter volumes, voxel-based morphometry analysis was conducted in Statistical Parametric Mapping (SPM8) [36] using the VBM8 toolbox (http://dbm.neuro.uni-jena.de/vbm). We utilized the optimized VBM process [37] which included 1) segmentation and extraction of the brain in native space, 2) normalization of the images to a standard space using a customized pediatric template, created via Template-O-Matic software [38] using images from all subjects, 3) segmentation and extraction of the normalized brain (extraction is repeated to ensure that no non-brain tissues remain), 4) modulation of the normalized images to correct for tissue volume differences due to the normalization procedure, and 5) inspection of the resulting gray matter images by expert raters, blinded to group assignment for quality, guided by boxplots and covariance matrices output by the VBM8 toolbox.

### Anatomical Parcellation

We generated 90 cortical and subcortical regions of interest (ROIs), excluding the cerebellum, from the Automated Anatomical Labeling (AAL) atlas using the WFU PickAtlas Toolbox [39]. The ROIs were identical to those used in previous graph analysis studies of structural and functional correlation networks [9], [17], [19], [20], [40]–[50]. These AAL ROIs were resliced to the same dimension as that of tissue segmented images obtained from the VBM preprocessing step. The ROIs were subsequently used to mask the individual modulated, normalized GM images and extract the average volume within each ROI using the REX toolbox (http://web.mit.edu/swg/software.htm). A linear regression analysis was performed at every ROI to remove the effects of age, gender and total brain volume. The residuals of this regression were then substituted for the raw ROI volume values [2], [5], [17], [19], [20] and are referred to as corrected regional gray matter volumes (RGV), hereafter.

### Network Construction

For each group, a 90 × 90 association matrix was generated by performing Pearson correlation coefficient between the corrected RGV across subjects [1]–[6], [17], [19], [20]. Thresholding the association matrices of different groups at an absolute threshold results in networks with a different number of nodes (and degrees) that might influence the network measures and reduce interpretation of between group results [32]. Therefore, binary networks are usually compared by thresholding the association matrices at fixed network densities (number of existing edges to the number of possible edges in the network). We derived binary adjacency matrices by thresholding the association matrices at a range of network densities (D_min_: 0.02∶0.5). The lower bound of the range is determined as the minimum density in which the networks of both groups are not fragmented (D_min_ = 0.22 (see Results section)). For densities above 0.5 the graphs become increasingly random (small-world index close to 1). Additionally, for anatomical networks, connections above this density are less likely biological [51]. Each of the derived binary adjacency matrices represents a network with a specific density.

### Null Networks

In order to estimate the small-world parameters of the constructed networks, three different choices of null networks were generated: 1) Null networks with the same number of nodes, total edges, and degree distribution as the network of interest (TOP) [30], [31]. This method preserves the degree distribution of the original network while randomizing its topology. There are several algorithms for generating random graphs with prescribed degree distribution [52]–[54]. These algorithms differ in terms of the type of output network (connected vs. disconnected, simple vs. complex) as well as the implemented method (e.g. matching vs. switching) [52]. The null networks generated for benchmarking correlation networks should be simple (no loops or parallel edges) and connected. We used the algorithm implemented in Brain Connectivity Toolbox (BCT) [28] that generates connected simple random graphs with prescribed degree sequence by directly searching for rewirable edge pairs in the original network. 2) Null networks corresponding to null covariance matrices that are matched to the distributional properties of the observed covariance matrix using Hirschberger-Qi-Steuer algorithm (HQS) [33], [34]. Null networks generated in this way preserve the transitive structure of the original network. Since the empirical correlation values might not follow a normal distribution, the density of the generated null networks were matched the density of the network of interest. 3) Null networks generated from the correlation of randomized corrected RGV data (COR). This method is an intuitive way of generating null networks for benchmarking correlation networks by applying the same network construction procedure on the randomized corrected GMV data. This procedure involved randomizing the original corrected RGV data for each subject separately and then obtaining a null correlation matrix by performing Pearson’s correlation analysis between the randomized RGV data across subjects. It should be noted that this procedure is different from correlating a group of random vectors. Finally, a binary null network is extracted by thresholding the generated null correlation matrix at a correlation level that matches the density of the resultant binary null network to the density of the network of interest. While the implemented TOP algorithm ensures that the generated null networks maintain connectedness (no fragmentation in the network), there is no guarantee that the null networks generated from COR and HQS method would be connected.

### Network Metrics

We investigated the influence of choice of null network on small-world parameters including clustering coefficient (CC), characteristic path length (CPL), and small-world index (SW). These network measures were extracted using the codes developed in the Brain Connectivity Toolbox (BCT) based on the formulation described in [28]. The network and statistical analyses were performed using our in-house software, graph analysis toolbox (GAT) [19].

### Influence of Null Networks on Small-world Parameters

In order to investigate the influence of null networks on small-world parameters of a network, we quantified the small-world parameters for the HC network and compared them among different choices of null networks. Normalized clustering coefficient C_HC_/C_null_, normalized path length [L_HC_/L_null_, and small-world index SW_HC_ = [C_HC_/C_null_]/[L_HC_/L_null_] were quantified for each type of null networks, namely TOP, HQS and COR, separately. These metrics were quantified at each density step for the specified range of densities [0.22∶0.02∶0.5]. We also quantified the small-world parameters for the ALL network to examine if the patient network follows a small-world organization across different null models.

Since the algorithms used for generation of null networks are stochastic by nature, the generated null networks would be different when applied many times to the same network. Therefore, C_null_ and L_null_ were considered as the mean clustering coefficient and the characteristic path length of 20 null random networks [19]. For comparison purposes, we examined whether the replicability of the null networks would differ between different null models. We generated 50 sets of null networks each consisting of 20 null networks for the HC network thresholded at D_min_ using different null models. We then performed a one-way analysis of variance (ANOVA) to compare the mean network parameters (C_null_ and L_null_) between these sets for each null model. In addition, we compared differences in the dispersion of C_null_ and L_null_ among different null models using Levene’s test of variance for 20 null networks.

In addition, to analyze the influence of null networks on between-group differences in small-world parameters, we quantified the small-world parameters of the ALL network employing different null networks and then compared the results with those obtained for the HC network. In addition, we compared the original clustering coefficient and characteristic path length between HC and ALL networks. This comparison allowed us to investigate the similarity between the results of group differences in original network metrics and differences in the normalized metrics.

Finally, for the purpose of comparison only, we also computed the small-world parameters for networks thresholded at a range of correlation values (rather than network density). The resultant network parameters are regarded as absolute network metrics [6] and will be used to examine if the results are compatible with those obtained by thresholding networks at a range sparsity thresholds.

### Statistical Analysis

Each network metric extracted across the specified density range [0.22∶0.02∶0.5] is represented by a curve that depicts the changes in network metric as a function of network density (threshold). In order to compare these curves between groups (or among choice of null networks), functional data analysis (FDA) was performed [40], [55]. In summary, each network measure curve was treated as a function (*y = f(x)*) where *y* represents the graph metric value and *x* represents the connection density. In order to compare two network metric curves (between groups or null models), the area *A*, between the two curves (*y_2_* vs. *y_1_*) was computed by summing the differences between *y*-values of the two groups (or the two null models) at each value of x: *A = Σ_i_ |y_2_ (x_i_) – y_1_ (x_i_)|* [40]. The obtained *A* value will be regarded as the difference in FDA between two network metric curves, hereafter. While there are several methods for comparing curves using FDA [55], we employed a non-parametric permutation test as described below. The FDA analysis was performed using our in-house GAT software [19].

**Figure 1:**
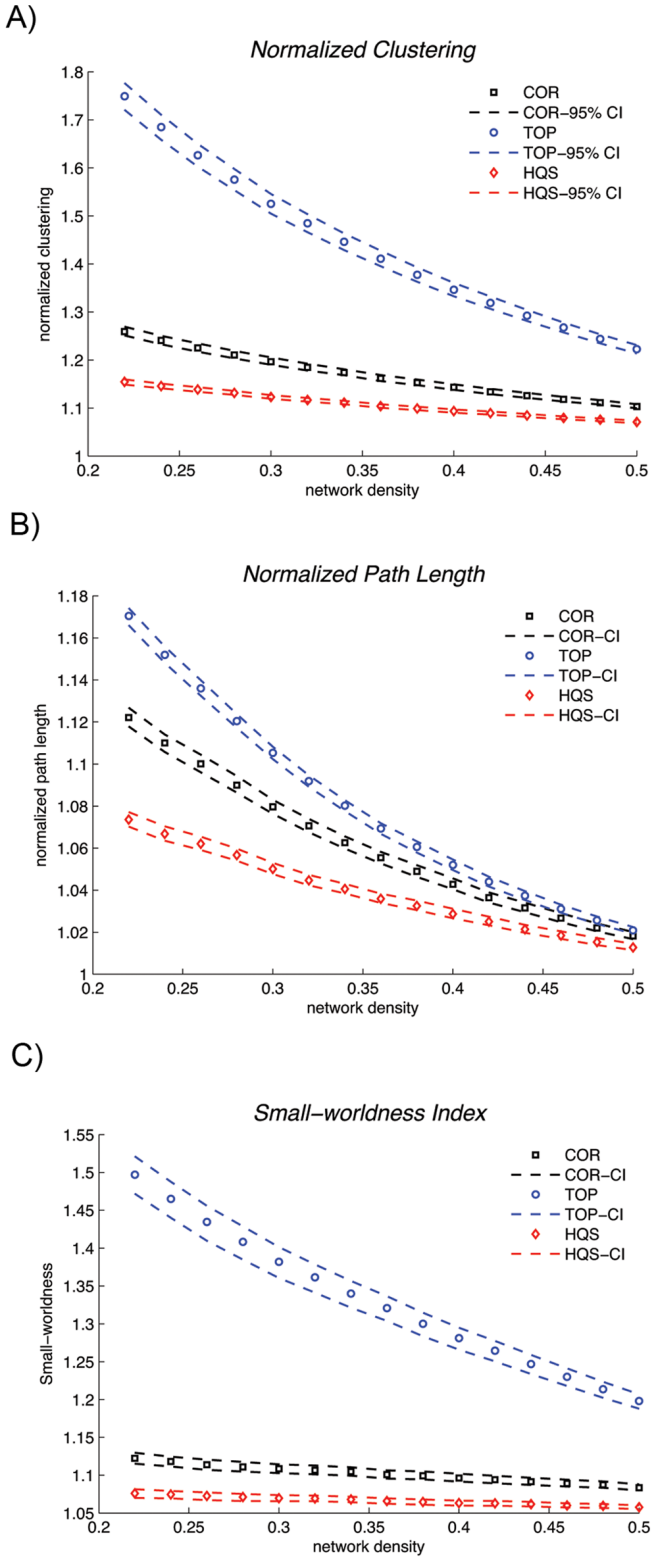
Changes in small-world properties of the HC bootstrap networks as a function of network density. A) normalized clustering, B) normalized path length and C) small-world index for different choices of null networks across the density range [0.22∶0.02∶0.5]. The dashed lines represent the 95% confidence interval for the mean network parameter of 300 bootstrap networks. All the benchmarking methods revealed a small-world organization for the HC networks.

**Figure 2:**
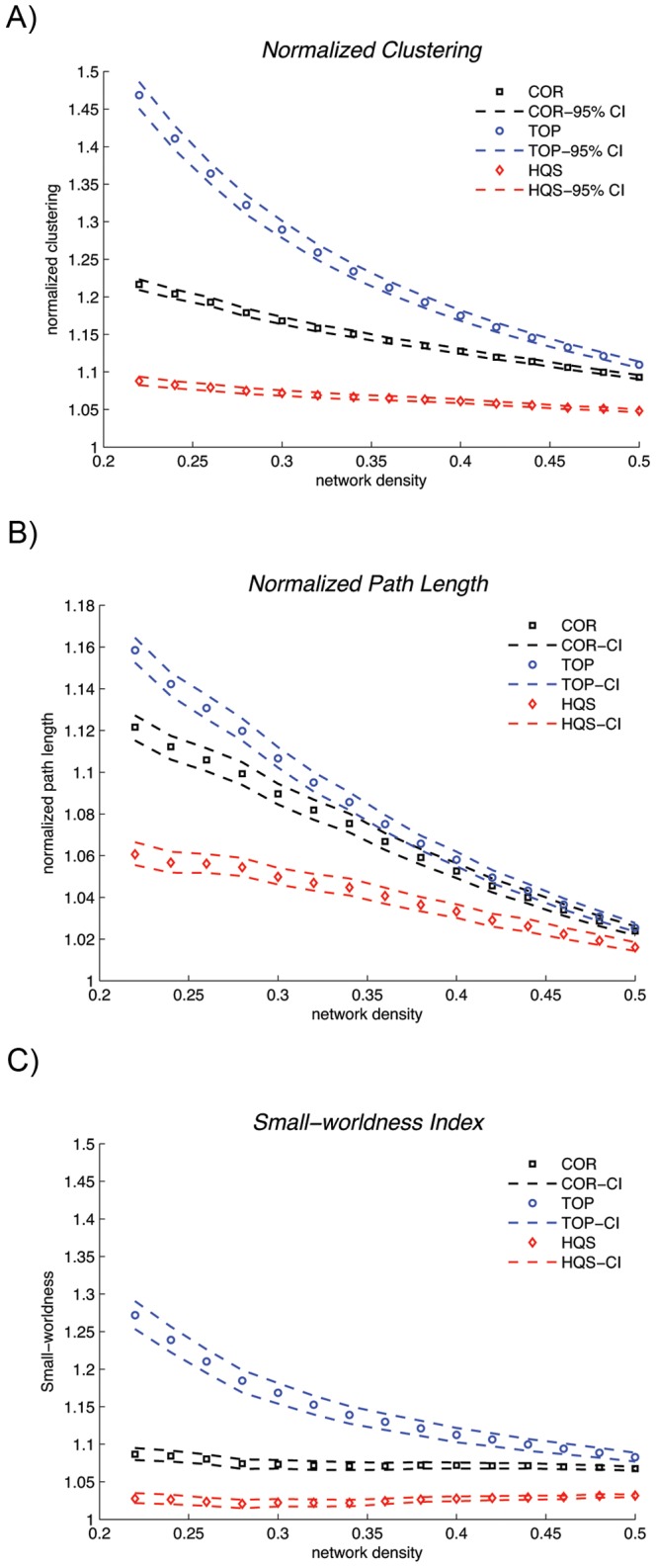
Changes in small-world properties of the ALL bootstrap networks as a function of network density. A) normalized clustering, B) normalized path length and C) small-world index for different choices of null networks across the density range [0.22∶0.02∶0.5]. The dashed lines represent the 95% confidence interval for the mean network parameter of 300 bootstrap networks. All the benchmarking methods revealed a small-world organization for the ALL networks.

In order to test the statistical significance of the differences in small-world parameter curves (SW_HC_), between different null networks, a non-parametric permutation test for dependent samples was performed as described in [16]. In summary, 1) 300 bootstrap samples of the association matrix of HC group were acquired by randomly selecting subjects’ corrected RGV data from HC group, with replacement, and computing the Pearson’s correlation coefficients. 2) The graph metric curves were quantified for each of the bootstrap samples and for each null network type. 3) The obtained curves were randomly shuffled between different null networks in each bootstrap sample across all samples. 4) The differences in FDA of the shuffled graph metric curves (i.e. the area between the two curves) among different null networks were calculated. 5) Steps 3–4 were repeated 5000 times and histograms of the between-null network differences in mean were constructed. 6) The observed actual between-null network differences in FDA were then placed on the constructed histograms and a p-value was calculated based on its percentile position. The permutation procedure was performed separately for each pair of null networks. It should be noted that sampling with replacement introduces an inflated correlation into the bootstrap correlation matrices. Thus, the mean of the network measures for bootstrap networks might not conform to those for original networks (Figure S1). However, this difference would not influence our results since we are comparing the null models across the same bootstrap networks (networks constructed from the same set of subjects). In addition, the obtained small-world parameters for bootstrap networks were consistent with previous reports (Figures 1 and 2).

To test the statistical significance of the differences in small-world parameters between groups, a non-parametric permutation test for independent samples with 1000 repetitions was used [2], [4], [8], [19], [20]. In each repetition, the corrected RGV data of each participant were randomly reassigned to one of the two groups so that each randomized group had the same number of subjects as in the original group. Then, an association matrix was obtained for each randomized group by performing Pearson’s correlation analysis. The binary adjacency matrices were then estimated by applying the same thresholding procedure as described above. The network metric curves were then calculated for all the constructed randomized networks. The differences in FDA of the network metric curves between randomized groups were then calculated resulting in a permutation distribution of difference under the null hypothesis. The actual between-group difference in FDA of the network metric curves was then placed in the corresponding permutation distribution and a p-value was calculated based on its percentile position.

We applied FDA on a cumulative threshold (cumulative FDA) over the full range of density [0.22∶0.02∶0.5] to investigate the influence of null network type on small-world properties of the correlation networks. In addition, we used a windowed thresholding procedure (windowed FDA) [40] to investigate the influence of different density regimes on small-world parameters quantified using different null networks. To this purpose, the specified density range [0.22∶0.02∶0.5] was divided into four threshold ranges [0.22∶0.02∶0.3], [0.3∶0.02∶0.38], [0.38∶0.02∶0.46] and [0.46∶0.02∶0.5] and the binary graphs were constructed by retaining connections that fell in each of these density ranges. It should be noted that in the windowed analysis, the connections within a lower-density window (e.g. [0.22∶0.02∶0.3]) would also present in higher-density windows (e.g. [0.3∶0.02∶0.38]). This procedure enables us to examine how adding lower-strength connections (i.e. less stable connections) to the network would affect the benchmarking results. The network metric curves were then compared across groups (and across choices of null networks) at each window, separately.

Finally, we examined whether the observed differences in network parameters between different null models are influenced by differences in the skewness of degree distributions of the networks. To this purpose, we performed a correlation analysis between skewness of the degree distribution of bootstrap networks across groups and differences in the obtained network metrics from different null models. We also performed correlation analysis between skewness of the degree distribution of bootstrap networks and the parameters of the corresponding null networks. These analyses were done on networks thresholded at D_min_ = 0.22.

## Results

### Influence of Null Networks on Small-world Parameters

Changes in small-world properties of the HC bootstrap networks as a function of network density [0.22∶0.02∶0.5] for different choices of null networks are shown in Figure 1. For all the null networks, the estimated normalized clustering coefficients of the HC structural correlation networks (CC_TOP_, CC_HQS_, CC_COR_) were greater than 1, the normalized path lengths (CPL_TOP_, CPL_HQS_, CPL_COR_) were close to 1, resulting in small-world indices (SW_TOP_, SW_HQS_, SW_COR_) that were greater than 1. The small-world indices were also greater than 1 in ALL bootstrap networks for different null models (Figure 2).

We also quantified the small-world parameters for the HC and ALL networks thresholded at a range of correlation values (Figures S2 and S3). The small-world indices quantified using different null models revealed a small-world architecture in both networks and confirms the results obtained by thresholding the networks at a range of sparsity thresholds.

The results of cumulative FDA analysis in the density range [0.22∶0.02∶0.5] and nonparametric permutation test for dependent samples showed that the cumulative FDA of normalized clustering coefficient, normalized path length, and small-world index in HC network are significantly different between all three choices of null networks (p<0.01) after correction for multiple comparisons (Bonferroni correction). The windowed FDA analysis also showed a significant difference in normalized clustering, normalized path length and small-world index of the HC network between all three null networks and for all the thresholding windows (p<0.01).

**Figure 3:**
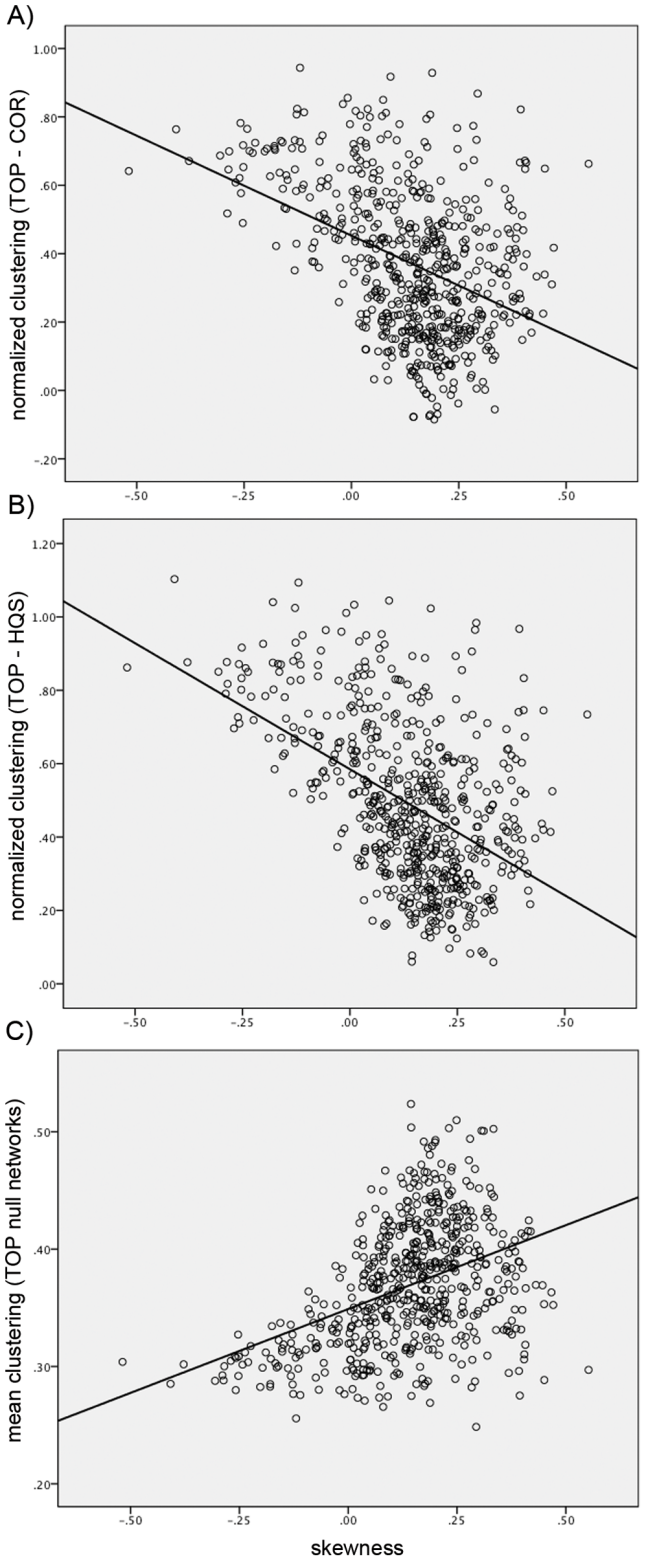
Relationship between skewness and normalized clustering. A significant negative correlation was found between the skewness of degree distribution of bootstrap networks (across both HC and ALL networks) and differences in normalized clustering between A) TOP and COR and B) TOP and HQS methods. C) the skewness of degree distribution of TOP null networks showed a significant positive correlation with mean clustering of corresponding networks.

The correlation analysis revealed a significant correlation between the skewness of degree distribution of bootstrap networks (across both groups) and the observed differences in normalized clustering between TOP and HQS (*r* = −0.44, p<0.01) as well as TOP and COR null models (*r = *−0.39, p<0.01) (Figure 3). Similar correlation results were observed between skewness and differences in small-worldness between TOP and HQS (*r* = −0.45, p<0.01) as well as TOP and COR models (*r* = −0.41, p<0.01). In addition, a significant positive correlation was found between the skewness of degree distribution of bootstrap networks and the clustering coefficient of corresponding TOP null networks (*r* = 0.41, p<0.01) (Figure 3C).

Comparing the replicability of null network parameters revealed no significant difference in the mean C_null_ and L_null_ between different sets (p>0.2). However, the dispersions of C_null_ and L_null_ were significantly higher for HQS and COR compared with TOP null networks (p<0.05). Changes in the mean C_null_ and L_null_ as a function of number of generated null networks for different null models are shown in Figure S4.

### Influence of Null Networks on between-group Differences in Small-world Parameters

**Figure 4:**
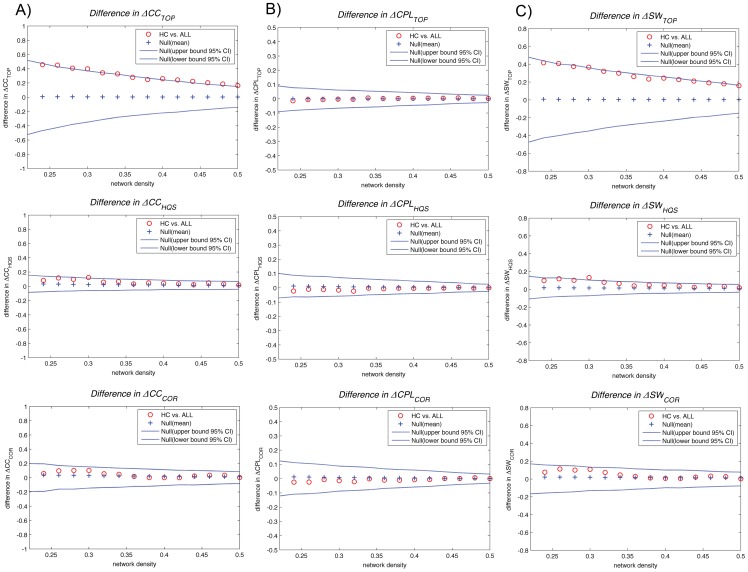
Between-group differences (HC vs. ALL) in small-world parameters. The 95% confidence intervals and between-group differences in A) normalized clustering (ΔCC_TOP_, ΔCC_HQS_, ΔCC_COR_), B) normalized path length (ΔCPL_TOP_, ΔCPL_HQS_, ΔCPL_COR_) and C) small-world index (ΔSW_TOP_, ΔSW_QS_, ΔSW_COR_) as a function of network density for different benchmarking methods. The red circles show the difference between HC vs. ALL networks; the circles falling out of the confidence intervals (blue dashed lines) indicate the densities in which the difference is significant. The positive values indicate HC>ALL and negative values indicate HC<ALL.

We also investigated the influence of null networks on the results of between-group differences in small-world parameters. Between-group differences (HC vs. ALL) in normalized clustering (ΔCC_TOP_, ΔCC_HQS_, ΔCC_COR_), normalized path length (ΔCPL_TOP_, ΔCPL_HQS_, ΔCPL_COR_) and small-world index (ΔSW_TOP_, ΔSW_QS_, ΔSW_COR_) as a function of network density are shown in Figure 4. The detailed results of nonparametric permutation test for independent samples on cumulative FDA and windowed thresholding data are given in Table 1. In summary, the cumulative FDA analysis over the density range [0.22∶0.02∶0.5] revealed that only normalized clustering quantified by the TOP method (ΔCC_TOP_) was significantly different between groups (p<0.05). The small-world indices derived from TOP methods (ΔSW_TOP_) were also marginally significant between groups (p = 0.056). The windowed thresholding procedure revealed that in lower densities (stronger correlation) [0.22∶0.02∶0.3], the small-world indices ΔSW_HQS_ was significantly different between groups (p<0.05) while ΔSW_TOP_ and ΔCC_TOP_ were only marginally significant (p = .058 and 0.05, respectively). On the other hand, in higher densities [0.38∶0.02∶0.46] and [0.46∶0.02∶0.5], only ΔCC_TOP_ was significantly different between groups (p<0.05).

**Table 1:** Significance of between-group differences in small-world parameters across different benchmarking methods.

	cumulative	w1	w2	w3	w4
***Clustering coefficient (HC>ALL)***					
COR	0.371	0.259	0.368	0.450*	0.401
HQS	0.151	0.089	0.152	0.255	0.262
TOP	0.046	0.050	0.052	0.043	0.035
Original	0.164	0.086	0.174	0.251	0.214
***Path length (HC>ALL)***					
COR	0.280*	0.251*	0.300*	0.366*	0453
HQS	0.284*	0.226*	0.284*	0424*	0.501
TOP	0.478*	0.404*	0.491	0.418	0.421
Original	0.357*	0.299*	0.373*	0.473*	0.447
***Small-world index (HC>ALL)***					
COR	0.247	0.127	0.247	0.481	0.415
HQS	0.076	0.047	0.082	0.272	0.223
TOP	0.056	0.058	0.063	0.063	0.058

The p-value of the permutation tests for Cumulative: [0.22∶0.02∶0.5], w1: [0.22∶0.02∶0.3], w2∶[0.3∶0.02∶0.38], w3: [0.38∶0.02∶0.46] and w4∶[0.46∶0.02∶0.5] density ranges.

* indicates that the network measure is greater in ALL than in HC.

**Figure 5:**
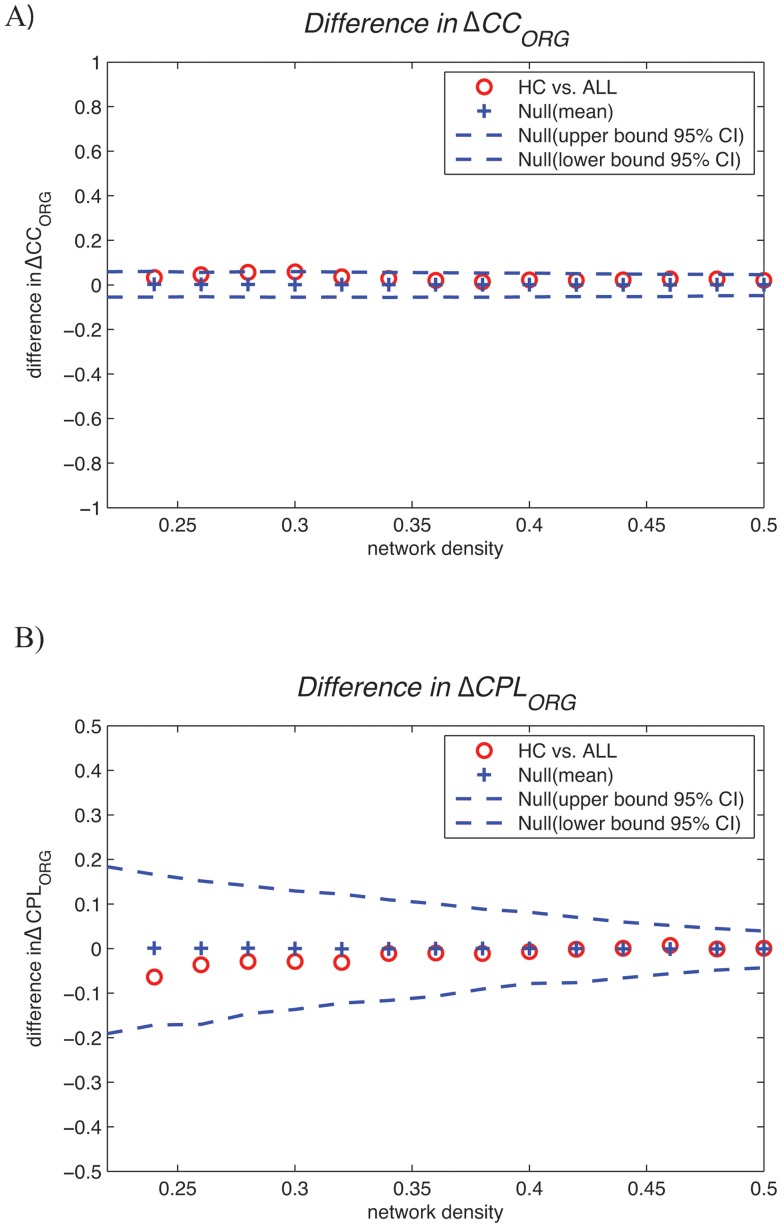
Between-group differences (HC vs. ALL) in original network parameters. The 95% confidence intervals and between-group differences in A) original network clustering (ΔCC_ORG_) and B) normalized path length (ΔCPL_ORG_) as a function of network density. The red circles show the difference between HC vs. ALL networks; the circles falling out of the confidence intervals (blue dashed lines) indicate the densities in which the difference is significant. The positive values indicate HC>ALL and negative values indicate HC<ALL.

Additionally, we compared group differences in original network measures, i.e. clustering coefficient (ΔCC_ORG_) and path length (ΔCPL_ORG_), between HC and ALL networks (Figure 5) (Table 1). Neither cumulative FDA nor windowed thresholding data reflected significant differences in original clustering and path length between groups.

## Discussion

Recent evidence suggests that brain networks constructed from correlations are inherently more clustered than random networks of the same size and degree. Thus, null networks constructed by randomizing topology are not a good choice for benchmarking small-world parameters of correlation networks. In the present report, we investigated the influence of choice of null networks on small-world parameters of gray matter correlation networks in healthy individuals (HC) and survivors of acute lymphoblastic leukemia (ALL). The results revealed that the choice of null network not only influences the estimated small-world parameters, it also influences the results of between-group differences in small-world parameters. Our data suggest that the choice of null network is quite crucial for interpretation of group differences in small-world parameters of structural correlation networks.

### Influence of Null Networks on Small-world Parameters

For all three choices of null models, the HC network showed a small-world architecture, i.e. the estimated normalized clustering coefficients of the network were greater than 1 and the normalized path lengths were close to 1 resulting in small-world indices of greater than 1 (Figure 1). However, the estimated small-world parameters were significantly different among the choices of null networks. As was expected, both the cumulative and windowed FDA results showed that CC_HQS_ and CC_COR_ were significantly lower than CC_TOP_. This is because the TOP method compared with HQS and COR, does not cancel out the effect of transitive structure induced by correlation transitivity. Thus, the HQS and COR methods give more conservative estimates of normalized clustering coefficient. On the other hand, CPL_HQS_ was significantly lower than CPL_TOP_ and CPL_COR_. This implies that the HQS method, compared with TOP and COR, underestimates the normalized path length of the network resulting in overestimation of network global efficiency [28], [53], [57]. Consequently, the HQS method gives a more conservative estimate of small-world index (SW_HQS_ <1.2) compared with TOP and COR methods. The same pattern was observed for small-world parameters in ALL network (Figure 2) as well as for networks thresholded at a range of correlation thresholds (Figures S2 and S3). Note that the rate of decrease in SW_HQS_ and SW_COR_ was much slower than the rate for SW_TOP_ for both ALL and HC networks. This is mainly influenced by the slower rate of decrease in normalized clustering in HQS and COR compared with TOP method. We speculate that correlation transitivity is mainly influenced by strong correlations and thus the amount of transitive clustering that cancels out at lower densities (strong connections) in HQS and COR methods is much higher compared with TOP method. However, as the lower strength connections are added (higher densities), less transitive structure is added to the network and thus the HQS and COR estimates of normalized clustering (and small-worldness) decrease at a slow rate.

Correlation analysis revealed that the skewness of degree distribution predicts the observed differences in normalized clustering and small-worldness between TOP and HQS and between TOP and COR models. Specifically, the skewness toward high-degree nodes reduced the differences in normalized network parameters between Top and other null models. We speculated that this difference might be driven by the influence of skewness on parameters of TOP null network. The results were confirmatory and the clustering coefficient of the TOP null network was higher for networks with skewness toward high-degree nodes. Since we did not observe such an effect for parameters of COR and HQS null networks, the differences in clustering coefficient between TOP and HQS as well as TOP and COR decreased for networks with skewness toward high-degree nodes. However, the mechanism underlying the observed positive correlation between skewness of original networks and clustering coefficient of corresponding TOP null networks remains unclear. The results suggest that networks with high skewness are less sensitive to correlation transitivity effect and the corresponding TOP null networks would be closer to HQS and COR null networks in terms of clustering.

While the replicability of null network parameters for 20 null networks were not significantly different within each null model, the dispersion of C_null_ and L_null_ for HQS and COR models was significantly higher than that for COR model. The observed difference in the dispersion remained significant even for 100 iterations of null networks. These data suggest that the mean C_null_ and L_null_ of 20 random networks gives a reliable estimate of small-world parameters for replicating the results for different null models. However, the network parameters in HQS and COR models would be more variable compared with those in TOP model.

### Influence of Null Networks on between-group Differences in Small-world Parameters

#### Cumulative FDA

The cumulative FDA results showed that normalized clustering is greater in the HC network than in ALL and normalized path length is greater in the ALL network than in HC, resulting in a small-world index that is greater in HC network. These results were consistent across all benchmarking methods. However, the choice of null network did influence the statistics of between-group differences in small-world parameters. The cumulative FDA analysis showed that ΔCC_TOP_ was statistically significant between groups while ΔCC_HQS_ and ΔCC_COR_ did not show significant difference between groups. This implies that the TOP method overestimates the differences in network clustering between groups compared with the HQS and COR methods. The estimated normalized clustering in HQS and COR methods as well as the statistics for ΔCC_HQS_ and ΔCC_COR_ were comparable suggesting that both HQS and COR methods are consistent for computing normalized clustering coefficient of structural correlation networks. Conversely, the TOP method gave the most conservative estimate of between-group differences in normalized path length among other methods while HQS method was the least conservative among them. The results of between-group differences in small-world index were more consistent between TOP and HQS methods than COR method. The COR method gave the most conservative estimate of between-group differences in small-world index among three methods. Together, the cumulative FDA result suggests that while the choice of null network influences the statistics of between group differences in small-world parameters, the direction of difference is preserved across all methods.

#### Windowed FDA

Consistent with cumulative FDA results, the windowed FDA results showed that the normalized clustering and small-world index were greater in the HC than in ALL network across all benchmarking methods and all windows. Normalized path length was greater in ALL than in HC network at lower densities (strong correlations) while it was smaller in ALL network at higher densities (weak correlations). This pattern was consistent across all methods except that the flip occurred in lower densities in TOP measures compared with HQS and COR measures. The observed flip in the direction of between-group differences in normalized path length at higher densities (weak correlations) can be attributed to increased randomized structure in both networks by introducing more weak densities. Perhaps, the TOP method is more susceptible to this random structure and therefore the flip occurs at lower densities when using this method.

In line with cumulative FDA results, the windowed FDA results suggest that the choice of null network influences the statistics of between-group differences in small-world parameters. For lower densities (strong correlations), the observed between-group difference in normalized clustering was the largest in TOP method among others. Conversely, the observed group difference in normalized path length was the smallest in TOP method. These results were consistent with cumulative FDA results. However, at higher densities where the network behavior becomes more random, the observed consistency was violated.

The pattern of group-differences in small-world index for windowed FDA was consistent with those obtained from cumulative FDA at higher densities [0.3 to 0.5]. At lower densities [0.22 to 0.3], the small-world index was significantly lower in the ALL network than in HC network for HQS method while it was nonsignificantly lower in ALL for TOP and COR methods. This suggests that the TOP and COR methods, compared with HQS, gives a less conservative estimate of between-group differences in small-world index for strong correlations. Together, the windowed FDA result confirms the cumulative FDA results by showing that the choice of null network influences the statistics of between group differences in small-world parameters. In addition, it showed that the choice of null network influences the direction of difference in normalized path length between groups.

Small-world parameter is a relative measure and one may argue that the drawbacks/advantages of each null model may affect the networks of both groups equally. However, our data showed that the choice of null model influences the direction of group differences in network measures. This is especially problematic since, for some null models, the network measures are higher in one group but are lower in the same group using a different null model. Therefore, our data emphasize the importance of a universal null model for benchmarking correlation networks.

### Which Null Network is More Suitable?

So far, we investigated how the choice of null network affects the small-world parameters of correlation networks at within-group and between-group levels using cumulative and windowed FDA. However, the critical question remains: which of the above mentioned null networks is more suitable for the purpose of benchmarking structural correlation networks?

Recently, Zalesky and colleagues [33] suggest that HQS method is more suitable for estimation of normalized clustering coefficient of correlation networks compared with TOP method. Unlike TOP method that annihilates the transitive structure of correlation networks, the HQS method does not affect the transitive structure and thus gives a more conservative estimate of normalized clustering for correlation networks. Using empirical data, our results confirmed that TOP method, compared with HQS, gives a higher estimate of the clustering coefficient of structural correlation networks. Thus, compared to TOP, HQS method is more appropriate for estimation of normalized clustering coefficient of correlation networks. However, the degree distribution of null networks generated using HQS method does not match the degree distribution of original network [33]. Thus, HQS method is less appropriate for estimation of normalized path length compared with TOP.

**Figure 6:**
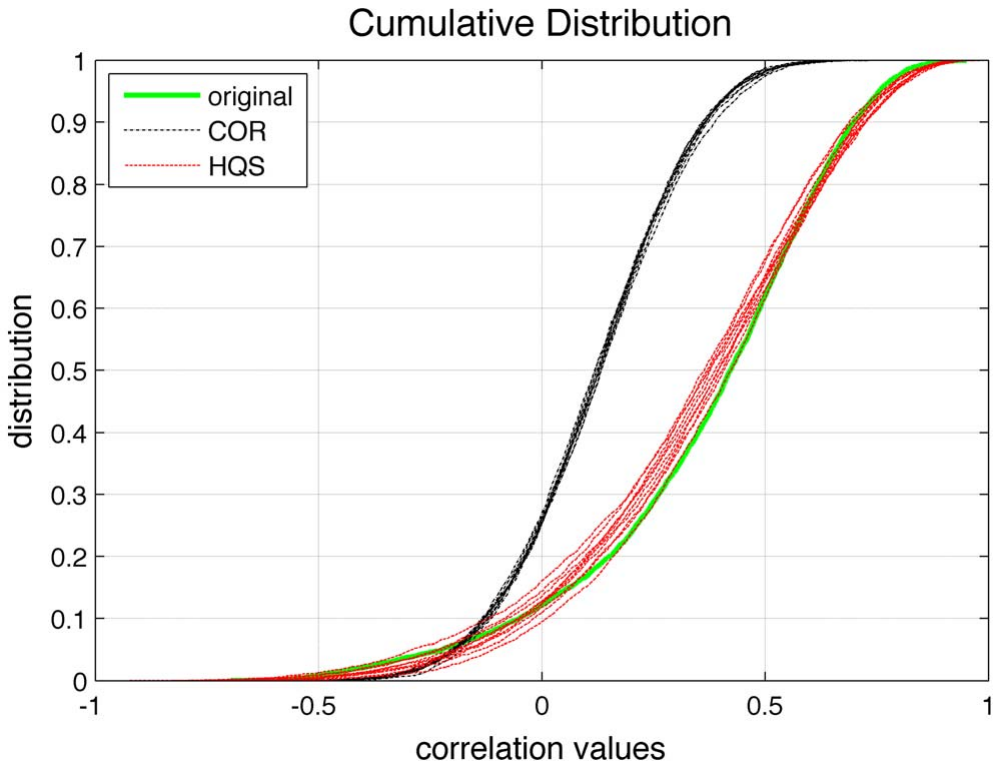
Cumulative distribution of correlation values. The correlation distribution of HC network (green), ten HQS null networks (red) and ten COR null networks (black). The cumulative distribution of correlation values in HQS model is very close to the original distribution while the distribution in COR model does not fit the original distribution. We did not show the correlation distribution for TOP model because the TOP model works on thresholded binarized networks.

Intuitively, the COR method should be a suitable method for normalization of clustering and path length since it applies the same network construction procedure on the randomized input data. However, careful examination of the correlation matrices generated using COR method reveals that the correlation distribution of the COR null network does not match the correlation distribution of the original network (Figure 6). This mismatch in correlation strength influences the correlation transitivity in the null models and further affects the clustering coefficient of the null networks [33]. Thus, the COR null networks also do not give an appropriate estimate of small-world parameters for correlation networks.

Apart from differences in the estimation of small-world parameters, the examined null models differ in terms of connectedness of the generated null networks. Unlike the TOP null networks, there is no guarantee that the outputs from HQS and COR methods would be connected. The connectedness of the null network is very crucial since the small-world parameters depend on the network size [32]. Therefore, fragmentation in the generated null networks would affect the normalized network metrics. However, this problem is slightly alleviated by averaging null network parameters across a number of null networks. In addition, disconnection of null networks at higher density thresholds is less probable. At lower densities, it is possible to discard null networks that are fragmented and only use the connected outputs for normalizing network parameters. Nonetheless, this process would increase the computational time and might not be feasible for all networks.

Together, our results suggest a lack of gold-standard null model for benchmarking correlation networks. Future research is needed to model gold-standard null networks that maintain both correlation distribution and degree distribution of the original correlation networks for appropriate normalization of clustering coefficient and characteristic path length of correlation networks, respectively. One potential solution is to use rewiring algorithms that work on weighted networks and preserve the degree (or strength) distribution of the original weighted network. These algorithms can be applied directly to correlation matrices. However, the available rewiring models for weighted networks only preserve the out-degree (or in-degree) distribution and thus not perfect for benchmarking correlation networks. Some attempts have been made to resolve this deficiency but the proposed models either fail to maintain the correlation distribution or fail to preserve the symmetry of the network [58], [59].

### Conclusions

We investigated the influence of choice of null networks on small-world properties of structural correlation networks. As was expected, the results revealed that the choice of null network significantly influences the estimates of small-world parameters of the networks, within group. In addition, the statistics of between group results were affected by the choice of null network. While the direction of the between-group differences in network parameters was not affected by the choice of null network at lower network density ranges (strong correlations), it was influenced at higher densities where the networks become more random. Finally, our data suggests that none of the available null models can be regarded as a gold-standard for benchmarking correlation networks and the relative strengths and weaknesses of the selected model should be carefully considered with respect to obtained network measures. Future studies need to examine new rewiring algorithms that work on weighted networks and preserve the degree (or strength) distribution of the original weighted network. Alternatively, hybrid models that employ correlation distribution from HQS method and apply the correlation values to the structure obtained from COR method are also promising.

Although we demonstrated the effects of null models on benchmarking small-world parameters for structural correlation networks, the results can be generalized to various kinds of correlation networks including networks constructed from gene expression and proteomics data [31], [60].

## Supporting Information

Figure S1 **Changes in the original clustering and path length of HC network as a function of network density.** A) clustering and B) path length for the original HC network (+) as well as the corresponding mean (SD) values for the HC bootstrap networks (squares). The mean network parameters for bootstrap networks were slightly deviated from those of the original network. Sampling with replacement results in having a number of similar subjects within the bootstrap samples that leads to obtaining inflated correlations and thus the results would deviate from those for original network.
(TIF)

Figure S2 **Changes in small-world properties of the HC network as a function of correlation threshold.** A) normalized clustering, B) normalized path length and C) small-world index for different choices of null networks as a function of correlation threshold. All the benchmarking methods revealed a small-world organization for the HC network. The pattern of differences in small-world parameters between null models is similar to the pattern observed for networks thresholded at a range of sparsity thresholds.
(TIF)

Figure S3 **Changes in small-world properties of the ALL network as a function of correlation threshold.** A) normalized clustering, B) normalized path length and C) small-world index for different choices of null networks as a function of correlation threshold. All the benchmarking methods revealed a small-world organization for the ALL network. The pattern of differences in small-world parameters between null models is similar to the pattern observed for networks thresholded at a range of sparsity thresholds.
(TIF)

Figure S4 **Changes in the mean C_null_ and L_null_ as a function of number of null networks generated.** A) Changes in the mean C_null_ for COR (top panel), TOP (middle panel) and HQS (bottom panel) null networks as a function of number of null networks generated. B) Changes in the mean L_null_ for COR (top panel), TOP (middle panel) and HQS (bottom panel) null networks as a function of number of null networks generated. No significant difference in the mean C_null_ and L_null_ were observed between different sets (p>0.2). However, the dispersions of C_null_ and L_null_ were significantly higher for HQS and COR compared with TOP null networks (p<0.05).
(TIF)
