# Supplementary figures and images for: Influence of Choice of Null Network on Small-World Parameters of Structural Correlation Networks
Source: PLoS One. 2013 Jun 28;8(6):e67354. doi: 10.1371/journal.pone.0067354 (PMC3696118; doi:10.1371/journal.pone.0067354)

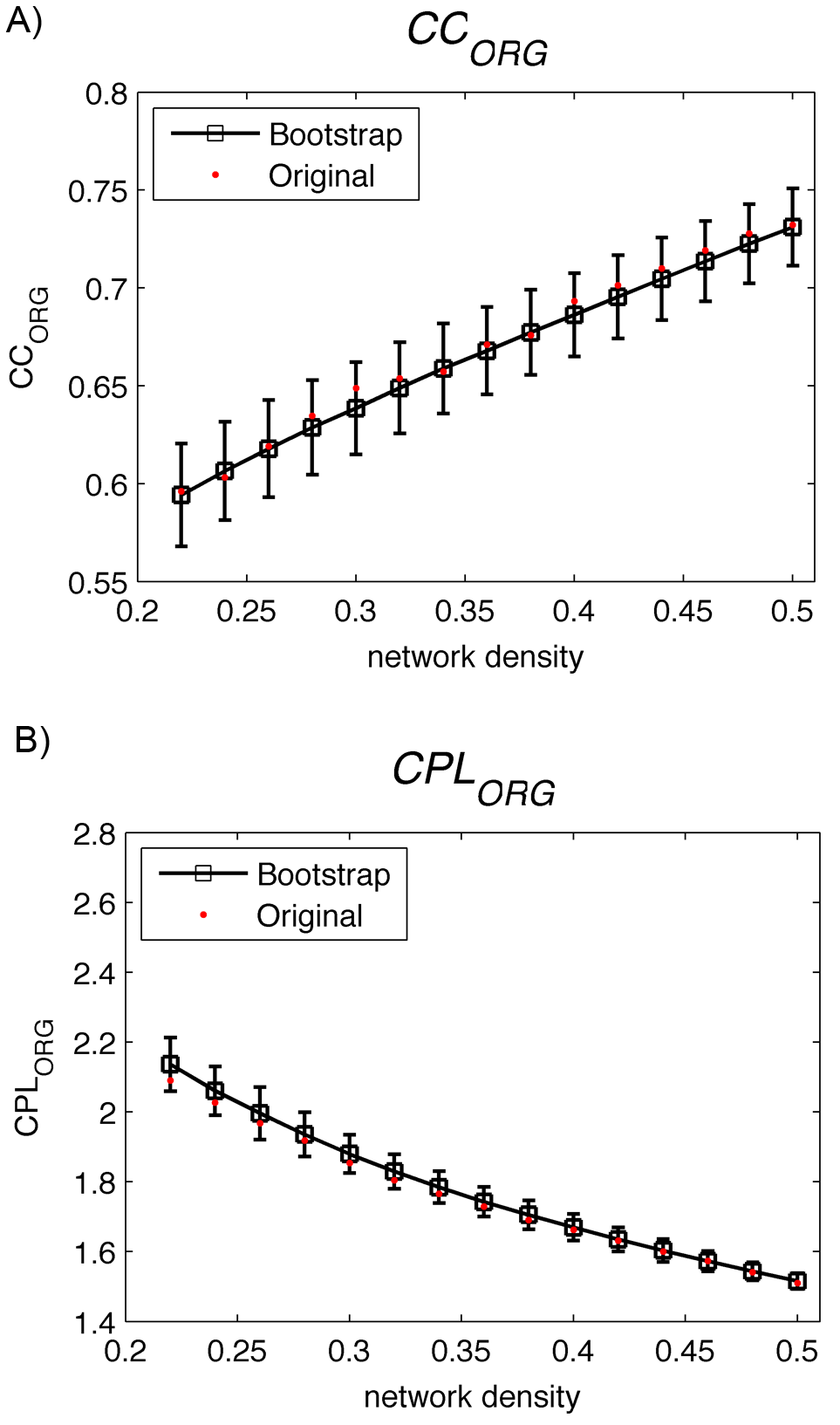

Supplement: Figure S1 — Changes in the original clustering and path length of HC network as a function of network density. A) clustering and B) path length for the original HC network (+) as well as the corresponding mean (SD) values for the HC bootstrap networks (squares). The mean network parameters for bootstrap networks were slightly deviated from those of the original network. Sampling with replacement results in having a number of similar subjects within the bootstrap samples that leads to obtaining inflated correlations and thus the results would deviate from those for original network. (TIF) [file pone.0067354.s001.tif]

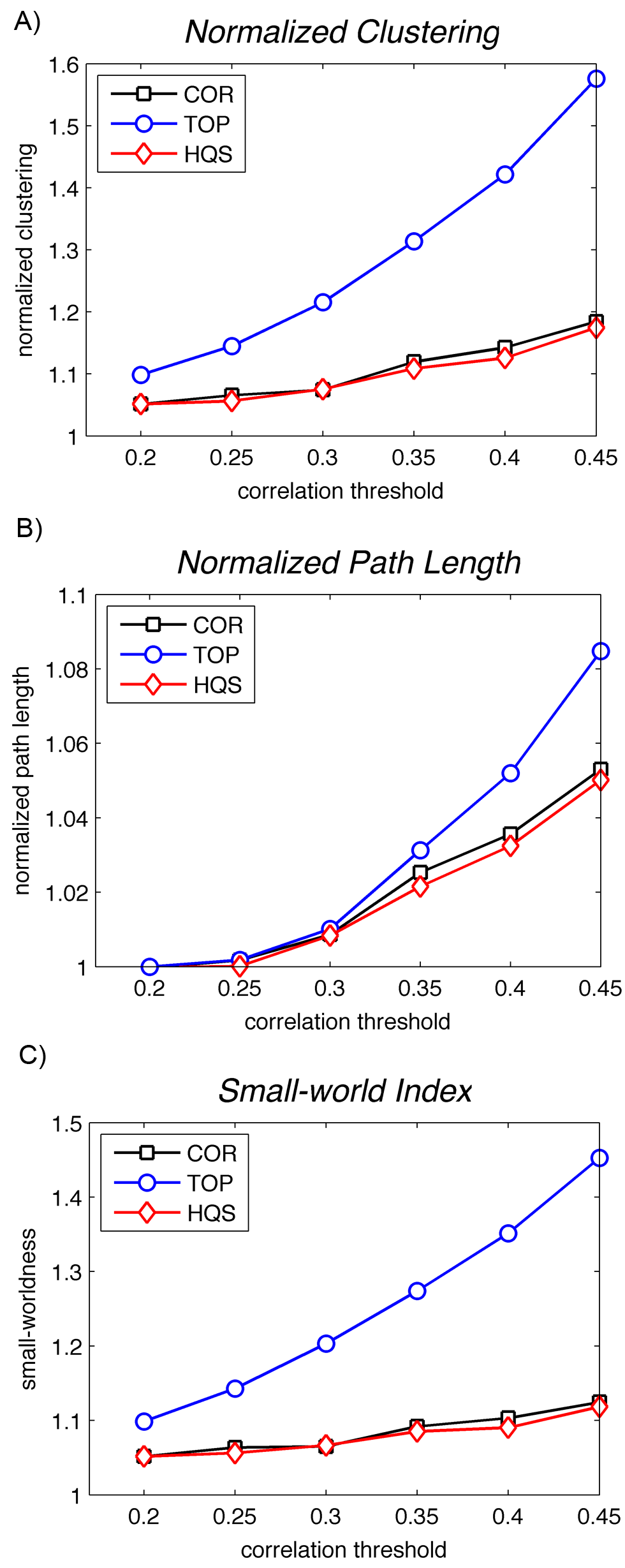

Supplement: Figure S2 — Changes in small-world properties of the HC network as a function of correlation threshold. A) normalized clustering, B) normalized path length and C) small-world index for different choices of null networks as a function of correlation threshold. All the benchmarking methods revealed a small-world organization for the HC network. The pattern of differences in small-world parameters between null models is similar to the pattern observed for networks thresholded at a range of sparsity thresholds. (TIF) [file pone.0067354.s002.tif]

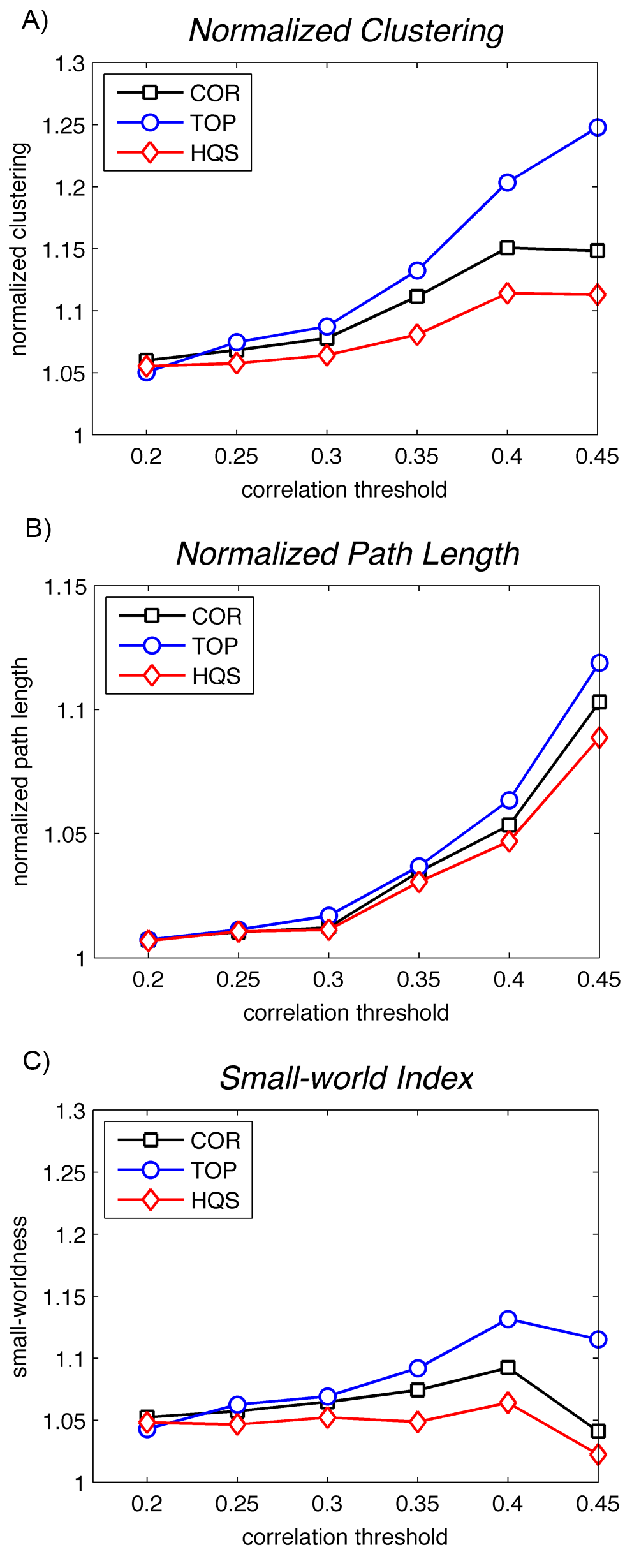

Supplement: Figure S3 — Changes in small-world properties of the ALL network as a function of correlation threshold. A) normalized clustering, B) normalized path length and C) small-world index for different choices of null networks as a function of correlation threshold. All the benchmarking methods revealed a small-world organization for the ALL network. The pattern of differences in small-world parameters between null models is similar to the pattern observed for networks thresholded at a range of sparsity thresholds. (TIF) [file pone.0067354.s003.tif]

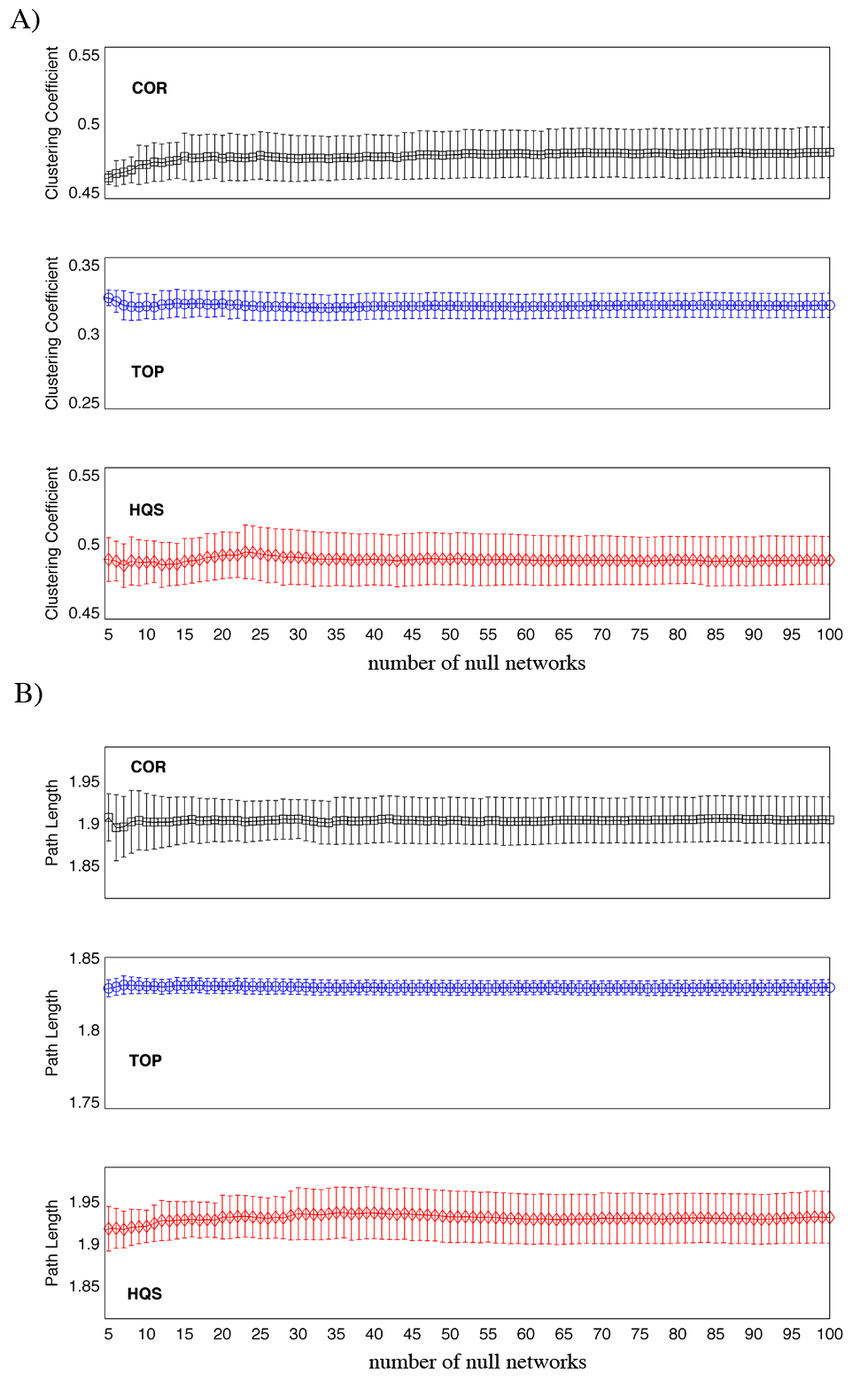

Supplement: Figure S4 — Changes in the mean Cnull and Lnull as a function of number of null networks generated. A) Changes in the mean Cnull for COR (top panel), TOP (middle panel) and HQS (bottom panel) null networks as a function of number of null networks generated. B) Changes in the mean Lnull for COR (top panel), TOP (middle panel) and HQS (bottom panel) null networks as a function of number of null networks generated. No significant difference in the mean Cnull and Lnull were observed between different sets (p>0.2). However, the dispersions of Cnull and Lnull were significantly higher for HQS and COR compared with TOP null networks (p<0.05). (TIF) [file pone.0067354.s004.tif]
